# Property Characterization and Photocatalytic Activity Evaluation of BiGdO_3_ Nanoparticles under Visible Light Irradiation

**DOI:** 10.3390/ijms17091441

**Published:** 2016-09-08

**Authors:** Jingfei Luan, Yue Shen, Lingyan Zhang, Ningbin Guo

**Affiliations:** State Key Laboratory of Pollution Control and Resource Reuse, School of the Environment, Nanjing University, Nanjing 210093, China; yueshen_sally@outlook.com (Y.S.); lingyanzhang2016@sina.com (L.Z.); ningbinguo@sina.com (N.G.)

**Keywords:** BiGdO_3_, solid state reaction, properties characterization, direct dyes, visible light irradiation, photocatalytic degradation

## Abstract

BiGdO_3_ nanoparticles were prepared by a solid-state reaction method and applied in photocatalytic degradation of dyes in this study. BiGdO_3_ was characterized by X-ray powder diffraction, X-ray photoelectron spectroscopy, scanning electron microscopy, Brunauer-Emmett-Teller, UV-Vis diffuse reflectance spectroscopy and transmission electron microscopy. The results showed that BiGdO_3_ crystallized well with the fluorite-type structure, a face-centered cubic crystal system and a space group *Fm3m 225*. The lattice parameter of BiGdO_3_ was 5.465 angstrom. The band gap of BiGdO_3_ was estimated to be 2.25 eV. BiGdO_3_ showed a strong optical absorption during the visible light region. Moreover, the photocatalytic activity of BiGdO_3_ was evaluated by photocatalytic degradation of direct dyes in aqueous solution under visible light irradiation. BiGdO_3_ demonstrated excellent photocatalytic activity in degrading Direct Orange 26 (DO-26) or Direct Red 23 (DR-23) under visible light irradiation. The photocatalytic degradation of DO-26 or DR-23 followed the first-order reaction kinetics, and the first-order rate constant was 0.0046 or 0.0023 min^−1^ with BiGdO_3_ as catalyst. The degradation intermediates of DO-26 were observed and the possible photocatalytic degradation pathway of DO-26 under visible light irradiation was provided. The effect of various operational parameters on the photocatalytic activity and the stability of BiGdO_3_ particles were also discussed in detail. BiGdO_3_/(visible light) photocatalysis system was confirmed to be suitable for textile industry wastewater treatment.

## 1. Introduction

Photocatalytic degradation has emerged as an efficient method for purification and treatment of polluted water and air in recent years [1,2,3,4,5,6,7,8,9,10,11,12]. Compared with conventional methods including physical, chemical and biological processes, photocatalysis was considered to be the most efficient and have a market prospect for the degradation of persistent organic pollutants [13,14,15]. Therefore, research on all kinds of photocatalysts for efficient photocatalysis has become a hot subject. Titanium dioxide (TiO_2_) was widely accepted as one of the most promising photocatalysts owing to its high activity, low cost, non toxicity and chemical stability [16,17,18,19]. However, TiO_2_ could only absorb UV light and it was not responding to the visible light area, thus the efficiency of TiO_2_ was low for utilization of sunlight (4%) [20,21,22]. UV light occupies only 5% in the solar spectrum, while the visible light between 400 and 750 nm occupies 41%. If the UV light part and the visible light part of sunlight could be fully utilized at the same time, the light quantum efficiency would be greatly improved.

One major direction for preparing efficient visible light photocatalysts is the use of non-metallic elements such as N, S, C, etc. to replace the oxygen element of TiO_2_. Element doping could reduce the band gap of the materials to extend its responding area to the visible region of the spectrum, and improved the light quantum efficiency to some extent [23,24,25,26,27,28,29,30,31]. Another direction is the preparation of composite oxides and complex compounds, such as rGO/C-MoO_3_, Co_*x*_Zn_1−*x*_Fe_2_O_4_-rGO, Fe_3_O_4_@C/Cu, Fe_3_O_4_@CuO, WO_3_/ZnO, Pt/Au/WO_3_, Ti/ZnO-Cr_2_O_3_, ZnO/CdS/TiO_2_, CoO-TiO_2_, CuO/SnO_2_/TiO_2_, BiPO_4_/TiO_2_/g-C_3_N_4_, AgInS_2_, ZnS-AgInS_2_, Ag_2_Mo_4_O_13_, K_6_Nb_10.8_O_30_, Y_2_Sn_2_O_7_, Ca_2_Nb-_2_O_7_, Bi_2_GaTaO_7_, ZnO-T, and ZnO nano- and microneedles [32,33,34,35,36,37,38,39,40,41,42,43,44,45,46,47,48,49,50] which showed photocatalytic activity under visible light irradiation. These metal oxides as photocatalysts have relatively high photocatalytic activity because of their unique arrangement of electronic structure, charge transport characteristics and light absorption properties, which could generate photoexcited charge carriers and show remarkable stability under varying conditions [51]. Bismuth oxide (Bi_2_O_3_) is an important p-type semiconductor and has been extensively investigated for various applications owing to its unique optical and electrical properties [52,53,54,55,56,57]. The band gap of Bi_2_O_3_ (2.58 eV) is narrower than the band gap of TiO_2_ (3.2 eV), thus Bi_2_O_3_ has been investigated as a visible-light-driven photocatalyst. However, the rapid recombination of the photogenerated electron-hole pairs led to a low activity of Bi_2_O_3_ [58,59,60]. Moreover, many bismuth-based mixed oxides such as BiVO_4_, Bi_2_WO_6_, Bi_2_Ti_2_O_7_, Bi_12_TiO_20_, and In_2_BiTaO_7_ [61,62,63,64,65,66,67,68,69] have been reported to be excellent visible light photocatalysts. The Bi 6s^2^ lone pairs of electron might play an important role in these compounds [70,71].

Some previous reports have shown that Gd^3+^, a rare earth ion, S doped on the photocatalysts could greatly enhance the photocatalytic activity under visible light irradiation. Guo et al. [72] evaluated the photocatalytic activity of Bi_1−*x*_Gd_*x*_FeO_3_ (*x* = 0, 0.05, 0.1, and 0.15) for the photodegradation of rhodamine B and the acquired results showed that the low concentration of Gd doping below *x* = 0.1 could significantly increase the photocatalytic activity of the photocatalysts compared with the pure BiFeO_3_ nanopowders. Luo et al. [73] reported that Gd-doped porous Bi_2_O_3_ microspheres, accompanied with different Gd concentrations, 0%, 1%, 2%, 3% and 4%, prepared by a simple hydrothermal synthesis method, could degrade 95.7%, 98.2%, 97.1% and 91.1%, respectively of rhodamine B under visible light irradiation for 120 min, and also could degrade 97.0%, 99.3%, 98.1% and 80.5%, respectively, of methyl orange under visible light irradiation for 28 min, which showed higher photocatalytic activity than the pure β-Bi_2_O_3_ catalyst. The main effect of rare earth ion Gd^3+^ doping on the photocatalytic activity of the photocatalysts was that Gd^3+^ could trap photoelectrons as efficient scavengers, which decreased the recombination probability of the electron-hole pairs [73,74,75].

Previous works indicated that photoexcitation of an electron in an O_2p_ and Bi_6s_ hybrid orbital could lead to a charge transfer occurring to a d orbital of the other metal in the composite oxide [76,77]. Gd had one occupied 4d orbital in the ground state and Gd–Bi composite oxide might be responding to the visible light irradiation. In the present paper, BiGdO_3_ nanoparticles were synthesized by a solid-state reaction method and tested to be efficient for photocatalytic degradation of dyes. The structural, optical and photocatalytic properties of BiGdO_3_ were studied in detail. This work concentrated on the photocatalytic properties of BiGdO_3_ for photodegradation of direct dyes in aqueous solution under visible light irradiation. The effect of various operational parameters on the photocatalytic degradation efficiency was examined in detail. The stability of both material and performance of BiGdO_3_ as a visible light photocatalyst was also investigated by material characterization and the repeated photocatalytic degradation tests. Finally, the degradation intermediates of DO-26 were observed and the possible photocatalytic degradation pathway of DO-26 was studied.

## 2. Results and Discussion

### 2.1. Structural and Optical Properties

#### 2.1.1. XRD Analysis

**Figure 1:**
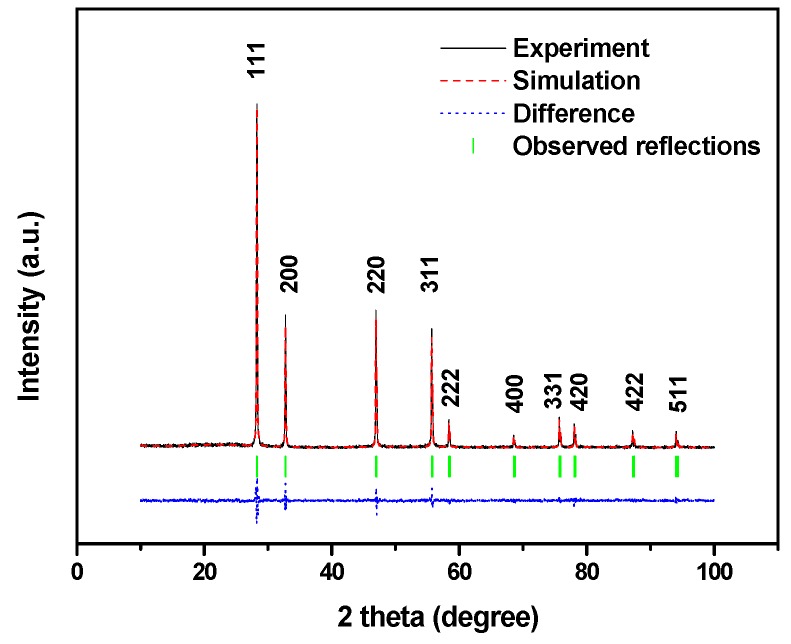
X-ray powder diffraction patterns and Rietveld refinements of BiGdO_3_. A difference (observed-calculated) profile is shown beneath. The tic marks represent reflection positions.

**Figure 2:**
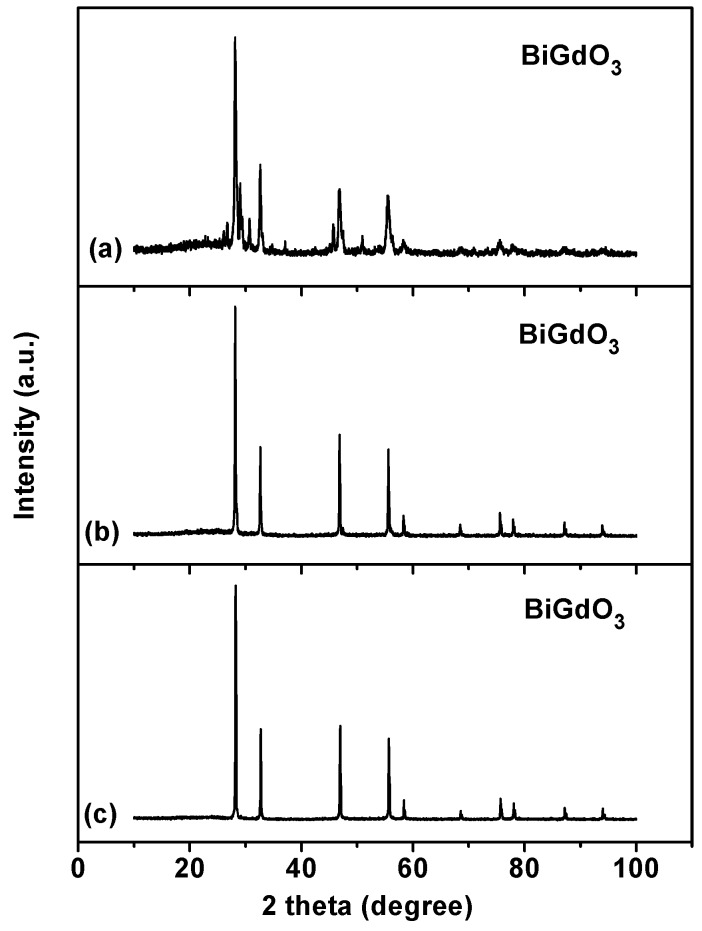
X-ray powder diffraction patterns of BiGdO_3_ samples prepared at different temperatures: (**a**) sample prepared at 750 °C for 10 h; (**b**) sample prepared at 1050 °C for 12 h; and (**c**) sample prepared at 750 °C for 10 h, 850 °C for 10 h and then at 1050 °C for 12 h with an intermediate regrinding process.

Figure 1 shows the powder X-ray diffraction pattern of BiGdO_3_ together with full-profile structure refinements of the collected data as obtained by the RIETAN™ [78] program, which was based on the Rietveld analysis. It can be seen in Figure 1 that all the diffraction peaks of the sample were sharp shape and were identical to the standard card (JSPDS 27-1043). It meant that the as-prepared bismuth gadolinium oxide BiGdO_3_ was single phase and was of high purity. According to the high purity of the precursors that were used in this study, it was unlikely that the observed space groups originate from the presence of impurities. Figure 2 shows the XRD patterns of BiGdO_3_ prepared at different temperatures. It was clear that BiGdO_3_ was not formed completely when BiGdO_3_ was treated at 750 °C for 10 h (as shown in Figure 2a) because there were small peaks which indicated that other phases existed. The reason was that the melting point 820 °C of Bi_2_O_3_ or the melting point 2330 °C of Gd_2_O_3_ was higher than 750 °C, as a result, the solid particle of Bi_2_O_3_ and the solid particle of Gd_2_O_3_ were difficult to form single phase BiGdO_3_ by mutual diffusion of the solid phase particles when they were put in the electric furnace with the highest sintering temperature of 750 °C. The characteristic diffraction peaks of the sample which was treated at 1050 °C for 12 h (as shown in Figure 2b) were not so clear compared with the sample which was treated at 750 °C for 10 h, 850 °C for 10 h, and then at 1050 °C for 12 h (as shown in Figure 2c) with an intermediate regrinding process. It showed that, with the three-step treatment procedure, the sintering reaction proceeded completely and a better crystallized BiGdO_3_ could be obtained. The main reasons were that the solid particle of Bi_2_O_3_ and the solid particle of Gd_2_O_3_ did not form single phase at the temperature of 750 °C, and then the grinding process increased the opportunity of high temperature diffusion between Bi_2_O_3_ and Gd_2_O_3_. Secondly, Bi_2_O_3_ melted and Gd_2_O_3_ did not melt at the temperature of 850 °C. In addition, high temperature diffusion between the molten liquid particle of Bi_2_O_3_ and the solid particle of Gd_2_O_3_ was easier to form single phase BiGdO_3_. Finally, the molten liquid particle of Bi_2_O_3_ and the solid particle of Gd_2_O_3_, which remained at the high temperature (1050 °C) for a long time could diffuse evenly and form a purer single phase BiGdO_3_. Moreover, rapid heating process avoided the formation of the single phase BiGdO_3_ in the heating intermediate process and guaranteed the single phase BiGdO_3_ to form beginning at 1050 °C. Furthermore, slow cooling process avoided the particles to become brittle because rapid cooling would result in imperfect crystallization of the single phase BiGdO_3_ or crystal defect of BiGdO_3_. In conclusion, the novelty of this method was that the synthesis of the purely single phase BiGdO_3_ utilized three-step sintering grinding method for the first time.

The crystal structure of BiGdO_3_ was successfully refined by using the Rietveld method from the X-ray diffraction data. The result of the final refinement for BiGdO_3_ displayed a good agreement between the observed intensities and calculated intensities. As a result, BiGdO_3_ possessed the fluorite-type structure, a face-centered cubic crystal system and a space group *Fm3m 225* (O atoms were included in the model). The lattice parameter α for BiGdO_3_ from the refined result was 5.465 angstrom. According to the Bragg Equation (1) and the calculation formula for cubic crystal face spacing Equation (2):

(1) 2dsinθ=λ=1.54056

(2) $$d=a/{\left(h^{2}+k^{2}+l^{2}\right)}^{1/2}$$

where *d* was the face spacing, θ was the diffraction angle, and *h*, *k* and *l* were the crystal indices. According to above equations, the lattice parameter α for BiGdO_3_ was calculated to be 5.472 angstrom, which was close to the refined result. All the diffraction peaks for BiGdO_3_ could be successfully indexed according to the lattice constant and above space group. The atomic coordinates and structural parameters of BiGdO_3_ are listed in Table 1. The outcome of the refinement for BiGdO_3_ generated the R-factor of the Rietveld refinement. *R*_P_ factor (unweighted residual error), one of the reliability index parameters, showed the difference between the observed and simulated powder diffraction patterns [79,80]. In our experiments, *R*_P_ was 14.14% with space group *Fm3m 225* for BiGdO_3_. This means that there was small difference between the observed and simulated powder diffraction patterns.

#### 2.1.2. XPS Spectra

**Figure 3:**
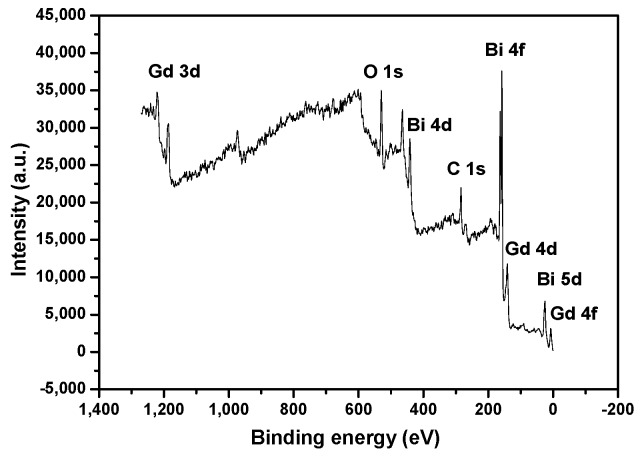
XPS spectrum of BiGdO_3_.

**Figure 4:**
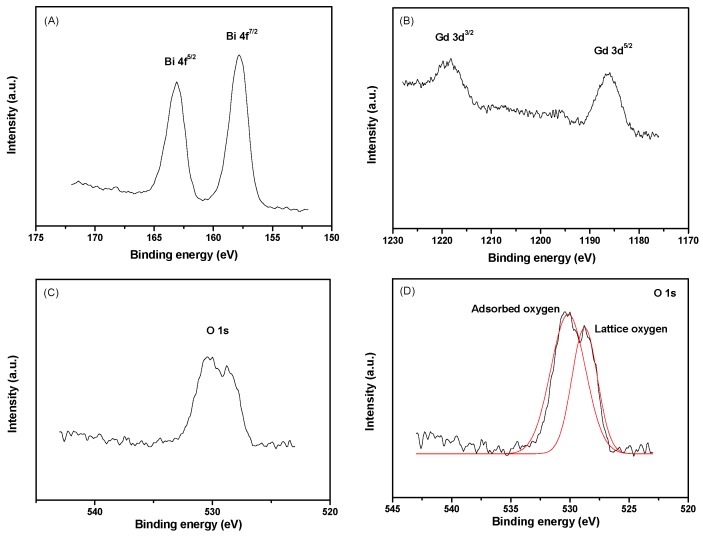
XPS peaks of: Bi 4f (**A**); Gd 3d (**B**); O 1s (**C**); and split of O 1s (**D**) in BiGdO_3_.

Figure 3 presents the XPS spectrum of BiGdO_3_. It was obvious that the observed XPS spectra of BiGdO_3_ showed neither shoulders nor widening peaks, suggesting (albeit not proving) the absence of any other phases. The sample showed the presence of bismuth (Bi 4f, Bi 4d, and Bi 5d), gadolinium (Gd 3d, Gd 4d, and Gd 4f), oxygen (O 1s) and carbon (C 1s). Moreover, the peak of C 1s was attributed to the surface adsorption pollution. Figure 4 shows the XPS peaks of: Bi 4f (A); Gd 3d (B); O 1s (C); and the split of O 1s (D) in BiGdO_3_. Various elemental peaks, which correspond to specific binding energies of BiGdO_3_, are provided in Table 2. The binding energies of Bi 4f^5/2^, Bi 4f^7/2^, Gd 3d^3/2^ and Gd 3d^5/2^ in BiGdO_3_ were 163.1, 157.8, 1218.2 and 1185.9 eV, respectively. It can be seen in Table 2 that there were two peaks with different binding energies for oxygen (O 1s). The split of O 1s peaks in Figure 4D was assigned to O 1s peaks of crystal lattice oxygen (528.8 eV) and surface adsorbed oxygen (530.4 eV) [81]. Surface adsorbed oxygen might correspond to the hydroxyl oxygen, which was significant for the improvement of the photocatalytic efficiency. Hydroxyl oxygen, which was adsorbed onto the surface of the photocatalyst, was in favor of the generation of hydroxyl radicals.

For BiGdO_3_, surface elemental analysis revealed that the average atomic ratio of Bi:Gd:O is 10.33:9.87:42.84. The ratio of Bi:Gd in the sample was almost the ratio of 1:1. The value of oxygen was high because there was a lot of adsorbed oxygen on the surface.

#### 2.1.3. TEM and SEM Analyses

**Figure 5:**
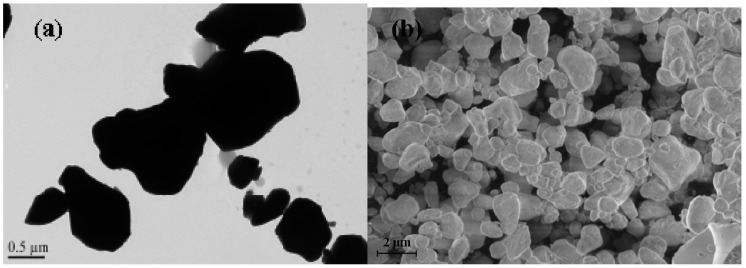
(**a**) TEM image of BiGdO_3_; and (**b**) SEM image of BiGdO_3_.

The morphology and microstructure of the as-prepared sample were examined by TEM, SEM and EDS. Figure 5 displays the TEM and SEM image of BiGdO_3_. We could observe from the images of BiGdO_3_ that the particles presented nanoscale and irregular-shaped appearance and that the distribution was relatively uniform. The average particle size of BiGdO_3_ approached 750 nm.

**Figure 6:**
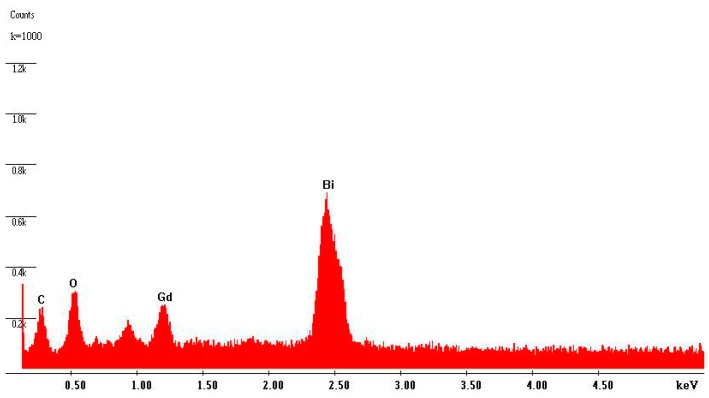
EDS spectrum of BiGdO_3_.

Figure 6 shows the EDS spectrum of BiGdO_3_. The EDS spectrum that was taken from the prepared BiGdO_3_ displayed the presence of bismuth, gadolinium and oxygen. Moreover, the peak of C was attributed to surface adsorption pollution. Other elements could not be identified from BiGdO_3_. The ratio of Bi:Gd:O in BiGdO_3_ sample was 12.57:12.88:39.53, which was accordant with the ratio of 1:1:3 expected from the BiGdO_3_ molecular formula. The results further indicated that the oxidation state of Bi, Gd and O ions from BiGdO_3_ were +3, +3 and −2, respectively. According to the above available data, it could be concluded that the sample which was prepared with Bi_2_O_3_ and Gd_2_O_3_ in a solid state reaction was BiGdO_3_, which was in agreement with the results of XRD and XPS analyses.

#### 2.1.4. BET Analysis

The specific surface area detected by the BET isotherm measurements was 3.25 m^2^·g^−1^ for BiGdO_3_. The specific surface area of BiGdO_3_ was almost 14 times smaller than that of TiO_2_, which was measured to be 46.24 m^2^·g^−1^. Many catalysts that are produced by solid state methods often exhibit low surface area, but might own good photocatalytic activity. Photocatalytic materials LiBi_4_M_3_O_14_ (M = Nb, Ta) that were prepared by Muktha et al. [82] showed reasonable photocatalytic activity for degrading dyes and organic compounds, despite their low BET surface area (0.3 or 0.1 m^2^·g^−1^).

#### 2.1.5. UV-Vis Diffuse Reflectance Spectra

**Figure 7:**
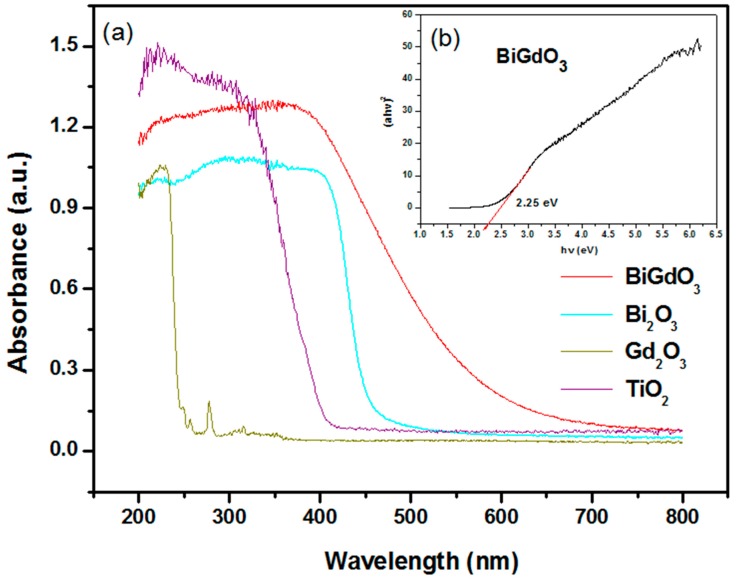
(**a**) UV-Vis diffuse reflectance spectra of BiGdO_3_, Bi_2_O_3_, Gd_2_O_3_ and TiO_2_; and (**b**) plot of (α*hν*)^1/*n*^ versus *hν* for BiGdO_3_ (inset).

UV-Vis diffuse reflectance spectroscopy was carried out to investigate the optical properties of the photocatalyst sample. Figure 7a presents the UV-Vis diffuse reflectance spectrum of BiGdO_3_, Bi_2_O_3_, Gd_2_O_3_ and TiO_2_. For a comparison, the UV-Vis diffuse reflectance spectra of Bi_2_O_3_, Gd_2_O_3_ and TiO_2_ were also provided in Figure 7a. As we all know, the well-known TiO_2_ whose absorption edge was at less than 380 nm had no response to the visible light irradiation. It could be seen that the absorption edge of BiGdO_3_ was found to be at 585 nm, which belonged to the visible region of the spectrum. It was obvious that the as-prepared BiGdO_3_ exhibited absorption range extending from the UV light region to 600 nm, which was wider than the range of Bi_2_O_3_. We could draw a conclusion from the researches reported by Oshikiri et al. [83] and Zheng et al. [84] that the positions and width of the conduction band and the valence band of BiGdO_3_ could be investigated by calculating the electronic band structure of Bi_2_O_3_, Gd_2_O_3_ and BiGdO_3_ with the plane-wave-based density functional method. Moreover, the band structure calculations of Bi_2_O_3_, Gd_2_O_3_ and BiGdO_3_ might be performed with the program of Cambridge serial total energy package (CASTEP) and first-principles simulation [85]. The CASTEP calculation was composed of the plane-wave pseudopotential total energy method according to the density functional theory. The valence band of Bi_2_O_3_ or Gd_2_O_3_ was composed of 2p orbital of oxygen, while the conduction band of Bi_2_O_3_ or Gd_2_O_3_ was composed of 6p orbital of bismuth or 5d orbital and 6s orbital of gadolinium, respectively. Our previous works similarly explained [85,86] that the conduction band of the BiGdO_3_ photocatalyst consisted mainly of Gd 5d orbital component, while the valence band of BiGdO_3_ consisted of mainly O 2p orbital component, Bi 6p orbital component and Gd 6s orbital component. The contribution of the valence band on the value of band gap of the BiGdO_3_ photocatalyst was mainly owing to the hybridization among Bi 6p orbital component, Gd 5d orbital component and Gd 6s orbital component. The top of the value band of BiGdO_3_ increased and the distance between the top of the valence band of BiGdO_3_ and the bottom of the conduction band of BiGdO_3_ decreased. This change caused the decrease of band gap of the BiGdO_3_ photocatalyst, which was the main reason for the red-shift of the spectrum for BiGdO_3_. Another possible reason was the different electronic structures of Bi_2_O_3_, Gd_2_O_3_ and BiGdO_3_.

For a crystalline semiconductor, the optical absorption near the band edge followed the equation [87,88]:

(3) $$\alpha h\nu =A{\left(h\nu -E_{g}\right)}^{n}$$

where A, α, *E*_g_ and *ν* were proportional constant, absorption coefficient, band gap and light frequency respectively. Within this equation, *n* determined the character of the transition in a semiconductor. *E*_g_ and *n* could be calculated by the following steps: (i) plotting ln(α*hν*) versus ln(*hν*-*E*_g_) by assuming an approximate value of *E*_g_; (ii) deducing the value of *n* based on the slope in this graph; and (iii) refining the value of *E*_g_ by plotting (α*hν*)^1/*n*^ versus *hν* and extrapolating the plot to (α*hν*)^1/*n*^ = 0. According to this method, the inset in Figure 7b shows the plot of (α*hν*)^1/*n*^ versus *hν* for BiGdO_3_. The band gap of BiGdO_3_ was calculated to be 2.25 eV according to above equation, while the value of *n* for BiGdO_3_ was 0.5, indicating that the optical transition for BiGdO_3_ is directly allowed. Furthermore, the band gap of Bi_2_O_3_ was calculated to be 2.65 eV. Thus, the above results indicated an enhanced visible light absorption of BiGdO_3_ and a narrower band gap compared with Bi_2_O_3_.

### 2.2. Evaluation of Photocatalytic Activity

The photocatalytic activities of the as-prepared BiGdO_3_ samples were evaluated by the degradation of Direct Orange 26, Direct Red 23 or phenol under visible light irradiation. Direct Orange 26, Direct Red 23 or phenol was degraded well by using BiGdO_3_ as a photocatalyst under visible light irradiation. The effect of various operational parameters, namely, catalyst loading, initial concentration of Direct Orange 26 or Direct Red 23, pH value, light intensity and coexisting ions on the photocatalytic degradation efficiency was investigated in detail.

#### 2.2.1. Photocatalytic Degradation of Direct Orange 26 or Direct Red 23 or Phenol

**Figure 8:**
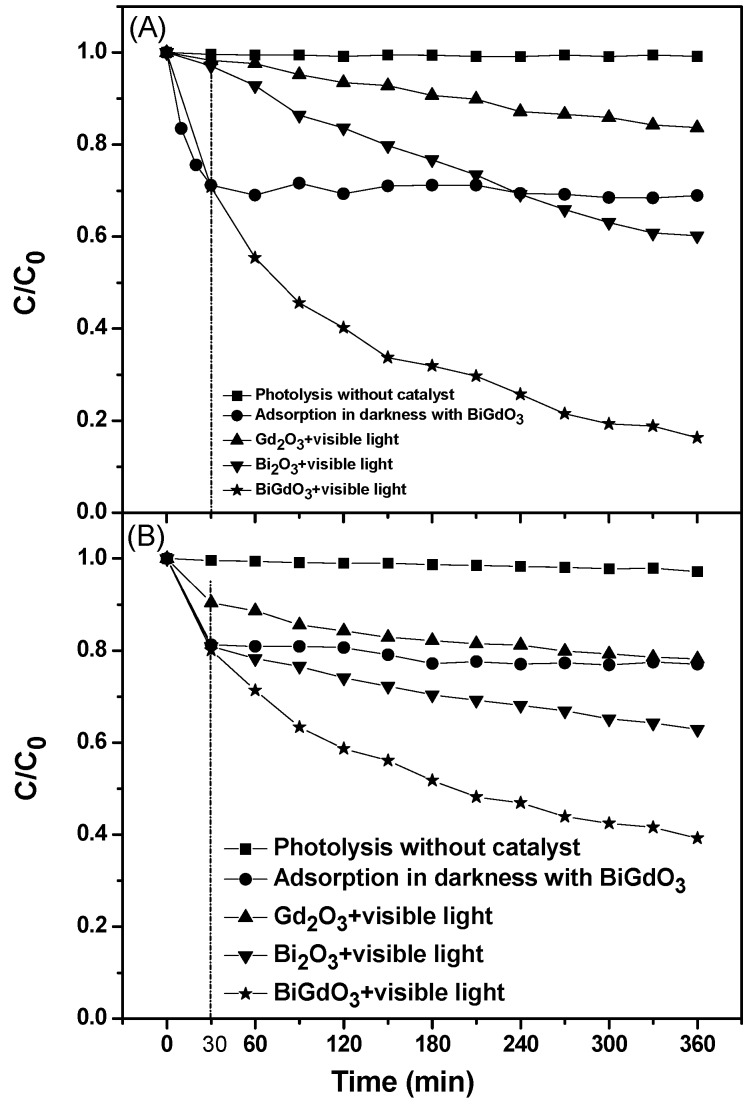
Comparison of removal efficiencies between Direct Orange 26 (**A**) and Direct Red 23 (**B**) under different conditions.

Figure 8 shows the comparison of removal efficiencies between Direct Orange 26 and Direct Red 23 under different conditions. As shown in Figure 8, the suspension had already reached adsorption/desorption equilibrium between the dye and the catalyst when stirred in the dark for 30 min, maintaining the decolorization rate of 31% for Direct Orange 26 (obtained from Figure 8A) and the decolorization rate of 23% for Direct Red 23 (obtained from Figure 8B) using BiGdO_3_ as a photocatalyst when stirred in the dark for 360 min. It could be concluded from the observation of the color of the catalyst that the adsorption process was just physical adsorption. The BiGdO_3_ particles changed to deep orange or red after the adsorption process while the particles still presented their original color after photocatalytic reaction, which meant that the adsorbed dye was also degraded during the reaction. It can be seen in Figure 8 that the photocatalytic degradation efficiency was 83.7% for Direct Orange 26 and 60.8% for Direct Red 23 using BiGdO_3_ as a photocatalyst under visible light irradiation within 360 min, while the photocatalytic degradation efficiency was 39.8% for Direct Orange 26 and 37.1% for Direct Red 23 using Bi_2_O_3_ as a catalyst or 16.3% for Direct Orange 26 and 21.8% for Direct Red 23 using Gd_2_O_3_ as a catalyst under visible light irradiation within 360 min. It can be seen in Figure 7a that Gd_2_O_3_ had no visible light response, but Gd_2_O_3_ did have a faint degradation efficiency on Direct Orange 26 or Direct Red 23 under visible light irradiation. The possible reasons of this phenomenon were the adsorption of the Gd_2_O_3_ sample and the photosensitive effect of Direct Orange 26 or Direct Red 23. These catalysts could be arranged in order of increasing degradation efficiency during the same reaction time: Gd_2_O_3_ < Bi_2_O_3_ < BiGdO_3_. The results in Figure 8 indicate that BiGdO_3_ had better photocatalytic activities for Direct Orange 26 or Direct Red 23 than Bi_2_O_3_ or Gd_2_O_3_. The cost of 100 g Gd_2_O_3_ and Bi_2_O_3_ was 63.2 dollars and 64.5 dollars, respectively, while the synthesis of 100 g pure BiGdO_3_ catalyst totally cost about 71 dollars, indicating that BiGdO_3_ had higher cost performance than Bi_2_O_3_ and Gd_2_O_3_. Additionally, there was almost no decrease for the UV-Vis absorbance signal of Direct Orange 26 or Direct Red 23 that was obtained under visible light irradiation in the absence of a photocatalyst.

**Figure 9:**
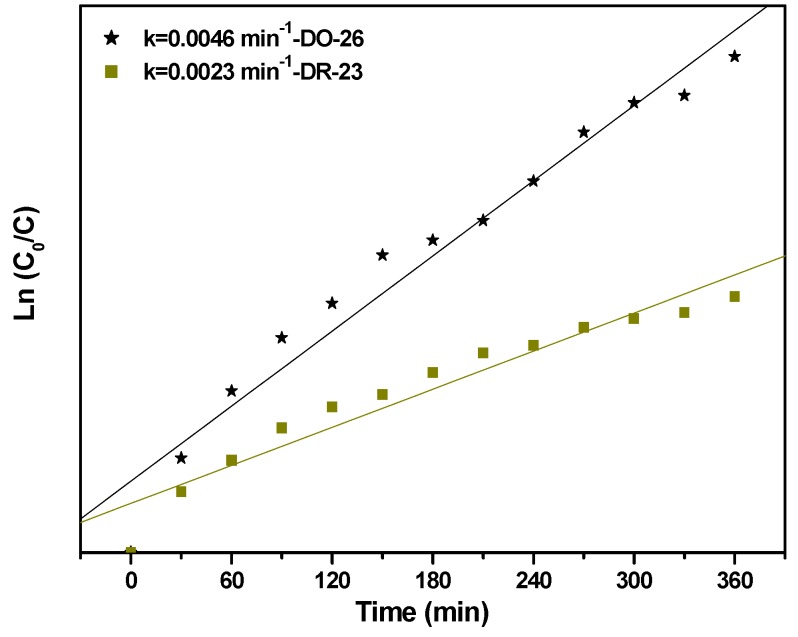
The first-order kinetic plots for the photocatalytic degradation of Direct Orange 26 or Direct Red 23 using BiGdO_3_ as a photocatalyst.

In order to quantitatively understand the reaction kinetics of the degradation of Direct Orange 26 or Direct Red 23, a pseudo first order was utilized to fit the experimentally obtained data. Figure 9 presents the reaction kinetics of the photocatalytic degradation of Direct Orange 26 or Direct Red 23 by using BiGdO_3_ as a photocatalyst. The pseudo first order indicated a linear correlation between ln(*C*_o_/*C*) and the visible light irradiation time. In the above equation, *C* represents the dye concentration at time *t*, and *C*_o_ represents the initial dye concentration. On the basis of the correlation result between ln(*C*_o_/*C*) and the irradiation time, the first-order rate constant *k* was estimated to be 0.0046 min^−1^ for Direct Orange 26. For Direct Red 23, the first-order rate constant *k* was estimated to be 0.0023 min^−1^, which was smaller than that for Direct Orange 26.

**Figure 10:**
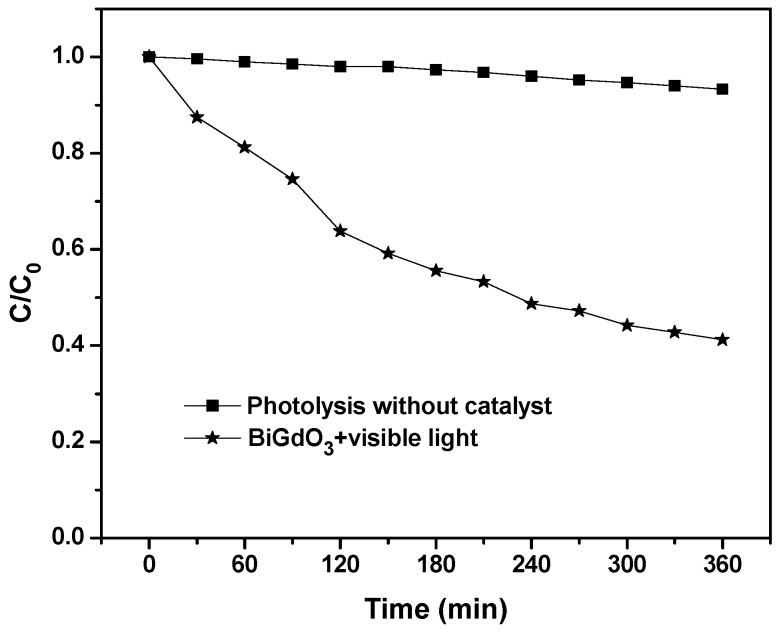
Photocatalytic activity of BiGdO_3_ for degrading phenol under visible light irradiation.

Figure 10 shows the degradation rate of phenol by using BiGdO_3_ as catalyst under visible light irradiation with respect to time. It can be seen in Figure 10 that the photocatalytic degradation rate of phenol was very low and could be ignored without adding BiGdO_3_ as a photocatalyst. Obviously, we could observe that an improved activity was also obtained when colorless phenol was also selected as a contaminant model with BiGdO_3_ as catalyst. The photocatalytic degradation efficiency of phenol was 59.8% by using BiGdO_3_ as a photocatalyst under visible light irradiation after 360 min, indicating that the catalyst BiGdO_3_ itself had photocatalytic activity and that the photodegradation process of Direct Orange 26 or Direct Red 23 by using BiGdO_3_ as a photocatalyst was not mainly due to photosensitive effect [89]. The above results revealed that the as-prepared catalyst BiGdO_3_ could decompose dye Direct Orange 26 or Direct Red 23 efficiently, at the same time, BiGdO_3_ could also degrade non-dye compounds, such as phenol. Ao et al. [90] used the same verification method to prove that the photodegradation process of dye by using as-prepared photocatalyst was not mainly due to the photosensitive effect.

#### 2.2.2. Effect of Various Operational Parameters

**Figure 11:**
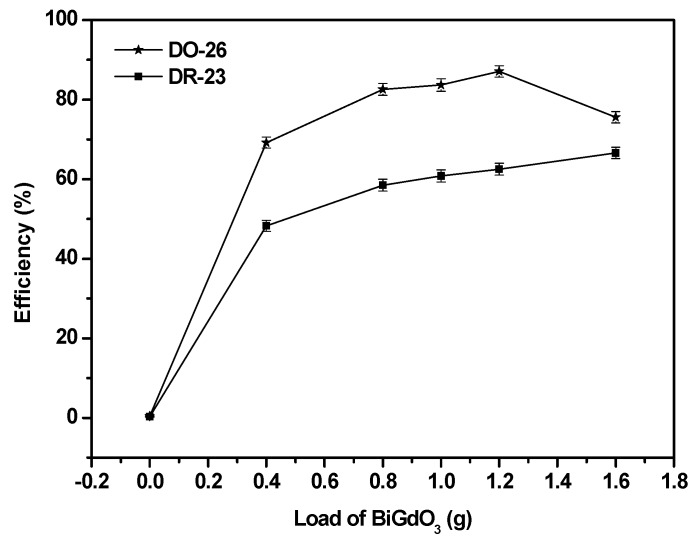
Effect of the catalyst loading on the photocatalytic degradation efficiency of Direct Orange 26 or Direct Red 23.

Figure 11 presents the effect of the catalyst loading on the photocatalytic degradation efficiency of Direct Orange 26 or Direct Red 23. The catalyst loading varied from 0.4 to 1.6 g/L with the initial concentration of 30 mg/L from Direct Orange 26 or Direct Red 23 and the original pH value of 6.2 from Direct Orange 26 or Direct Red 23. It can be seen in Figure 11 that the photocatalytic degradation efficiency of Direct Red 23 slightly increased while the catalyst loading increased from 0.4 to 1.6 g/L. The photocatalytic degradation efficiency of Direct Orange 26 increased while the catalyst loading increased from 0.4 to 1.2 g/L but decreased when the catalyst loading increased further to 1.6 g/L. The decrease of degradation efficiency for above dyes with increasing catalyst loading could be attributed to the increased opacity of the solution, which hindered the light transmission through the solution. Owing to the decrease of the effective light intensity, the photo generation of electrons and positive holes would be reduced and then the photocatalytic degradation efficiency of above dyes was also reduced [91,92]. With the catalyst loading of 1.2 g/L, the photocatalytic degradation efficiency of Direct Orange 26 was 87.1% which was a little higher than 83.7% with the catalyst loading of 1.0 g/L. Therefore, taking both the economic and efficient factors into consideration, the catalyst loading of 1.0 g/L was chosen as optimum loading content.

**Figure 12:**
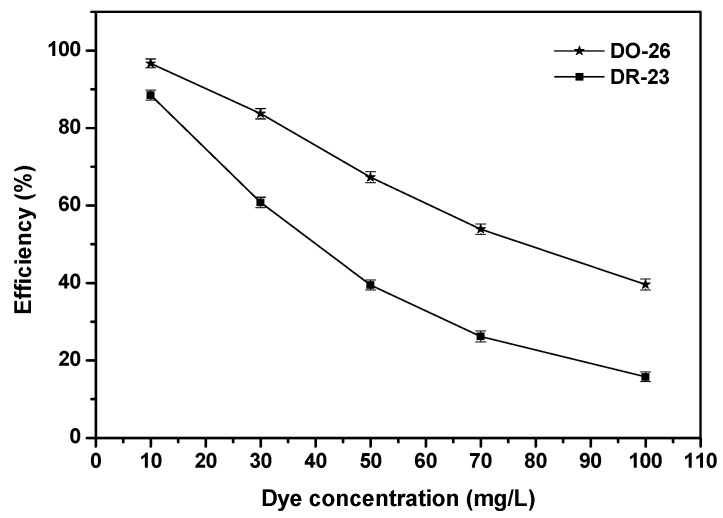
Effect of the initial concentration of Direct Orange 26 or Direct Red 23 on the photocatalytic degradation of Direct Orange 26 or Direct Red 23.

Figure 12 describes the effect of the initial concentration of Direct Orange 26 or Direct Red 23 on the photocatalytic degradation of Direct Orange 26 or Direct Red 23. With the initial catalyst loading of 1.0 g/L and the original pH value of 6.2 within the dye solution, the initial concentration of Direct Orange 26 or Direct Red 23 varied from 10 to 100 mg/L. It was found from Figure 12 that with increasing the initial concentration of Direct Orange 26 or Direct Red 23, the degradation efficiency of Direct Orange 26 or Direct Red 23 decreased. The main reason was that with increasing the concentration of above dyes, the amount of organic species which would be degraded increased [93], but the photocatalyst loading, the generation amount of e^−^/h^+^ pairs, dissolved oxygen concentration, intensity of light illumination and light illumination time were constant during the photocatalytic degradation process. Another reason was that the high concentration of Direct Orange 26 or Direct Red 23 reduced the light transmittance possibility towards the solution [94]. Thus, when increasing the initial concentration of Direct Orange 26 or Direct Red 23, the photocatalytic degradation efficiency of above dyes decreased. Comprehensively, considering the total pollutant removal efficiency and suitable reaction time, the initial concentration of 30 mg/L for Direct Orange 26 or Direct Red 23 was selected in the following reactions.

**Figure 13:**
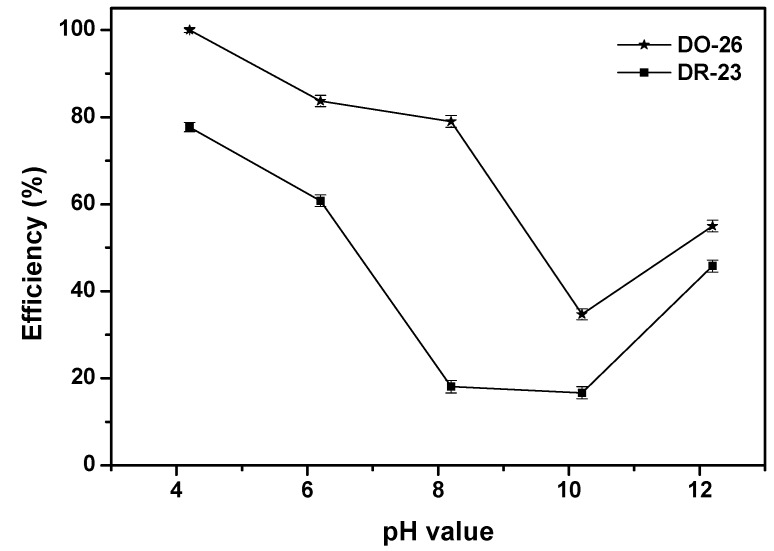
Effect of the initial pH value on the photocatalytic degradation efficiency of Direct Orange 26 or Direct Red 23.

Solution with the initial concentration of 30 mg/L for Direct Orange 26 or Direct Red 23 and catalyst loading of 1.0 g/L was utilized to investigate the effect of the initial pH value on the photocatalytic degradation efficiency of Direct Orange 26 or Direct Red 23. Figure 13 shows the effect of the initial pH value on the photocatalytic degradation efficiency of Direct Orange 26 or Direct Red 23. The initial pH value of Direct Orange 26 solution or Direct Red 23 solution varied from 4 to 12 by utilizing the solution of HCl or NaOH. As shown in Figure 13, the degradation efficiency of Direct Orange 26 or Direct Red 23 increased with decreasing pH value within acidic pH value range and the degradation efficiency of Direct Orange 26 or Direct Red 23 achieved the highest at the pH value of 4. During alkaline pH value range, with the increase of pH value, the degradation efficiency of Direct Orange 26 or Direct Red 23 firstly decreased and then increased. The minimum degradation efficiency of Direct Orange 26 or Direct Red 23 was found to be at the pH value of 10. The effect of the initial pH value was significantly important and generally complex in photocatalytic degradation of organic pollutants [81]. It was reported that the effect of the initial pH value on the photocatalytic degradation efficiency of organic pollutants performed mainly on surface adsorption, the aggregation of the catalyst and the band position of the catalyst in the solution [95,96]. The initial pH value could directly affect the nature of the charge, which was carried by the catalyst surface and the adsorption behavior of the pollutant on the catalyst surface. In addition, direct dyes were easily adsorbed on to the surface of the catalysts under strong acidic condition. Therefore, strong acidic condition was better for excellent photocatalytic degradation of direct dyes according to above results. Moreover, there were a lot of OH^−^ anions, which existed in the solution when the pH value was from 10 to 12. The h^+^ could easily react with a large amount of OH^−^ for generating active **·**OH species which could increase the photocatalytic degradation efficiency of Direct Orange 26 or Direct Red 23 [97,98,99,100]. Therefore, the degradation efficiency of Direct Orange 26 or Direct Red 23 could increase during strong alkaline pH value range.

**Figure 14:**
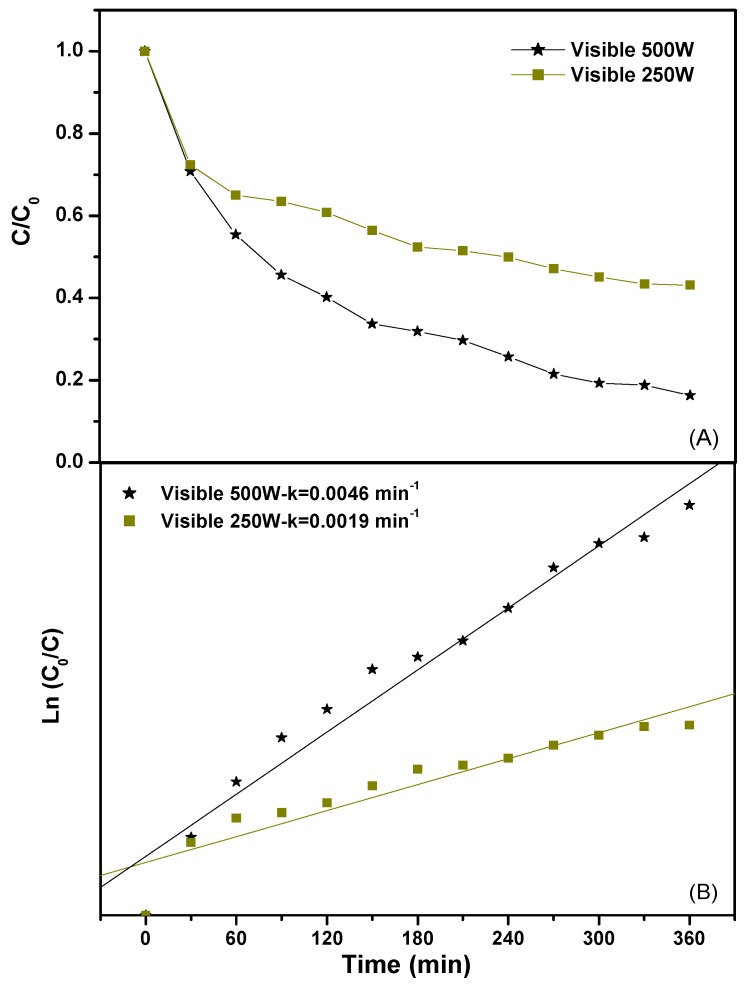
Effect of the light intensity on the photocatalytic degradation efficiency of Direct Orange 26 (**A**); and the first-order kinetic plots for the photocatalytic degradation of Direct Orange 26 (**B**) with different light intensity under visible light irradiation.

**Figure 15:**
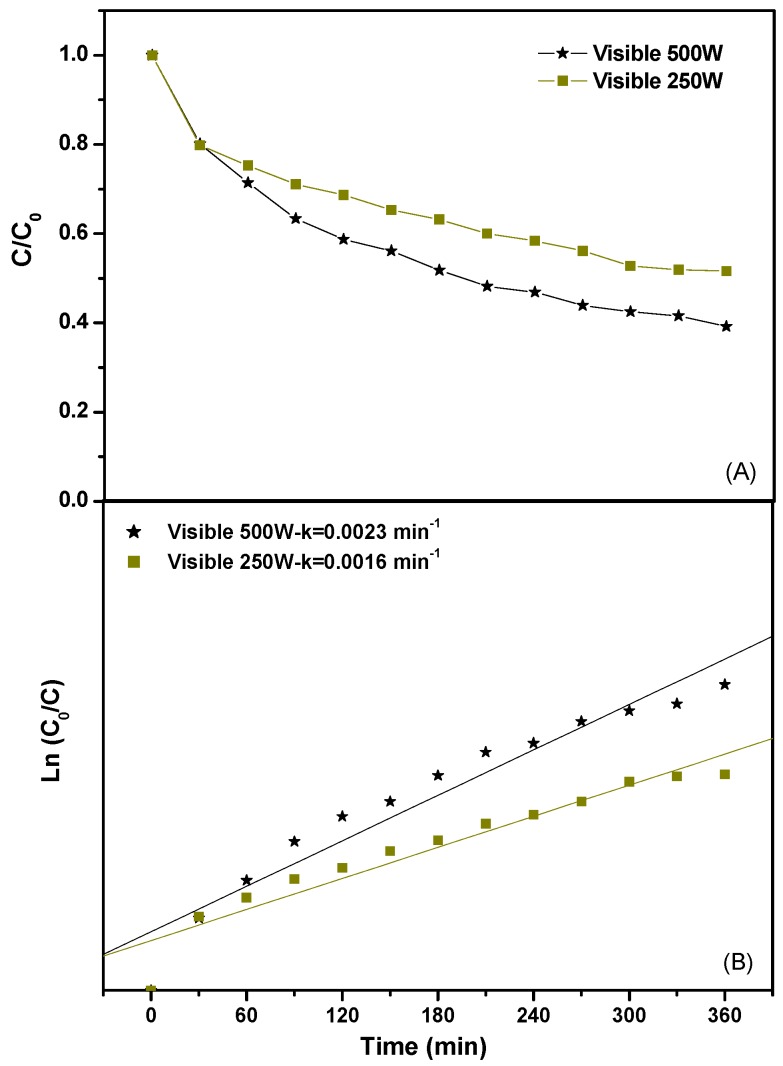
Effect of the light intensity on the photocatalytic degradation efficiency of Direct Red 23 (**A**); and the first-order kinetic plots for the photocatalytic degradation of Direct Red 23 (**B**) with different light intensity under visible light irradiation.

In order to investigate the effect of the visible light intensity on the photocatalytic degradation efficiency of Direct Orange 26 or Direct Red 23, two kinds of light source were applied in the reaction system. Figure 14 shows the effect of the light intensity on the photocatalytic degradation efficiency of Direct Orange 26 and the first-order kinetic plots for the photocatalytic degradation of Direct Orange 26 with different light intensity under visible light irradiation. As shown in Figure 14A, the photocatalytic degradation of Direct Orange 26 with a 500 W Xe arc lamp performed better than that with a 250 W Xe arc lamp. The photocatalytic degradation efficiency of Direct Orange 26 with a 500 W Xe arc lamp was 83.7% while that with a 250 W Xe arc lamp was only 56.9% after visible light irradiation of 360 min. The reaction kinetics of the photocatalytic degradation of Direct Orange 26 with BiGdO_3_ as a photocatalyst under different light irradiation conditions were clearly demonstrated in Figure 14B. According to the correlative result between ln(*C*_o_/*C*) and the light irradiation time, the first-order rate constant *k* was estimated to be 0.0019 min^−1^ with a 250 W Xe arc lamp as light source. While the first-order rate constant *k* was estimated to be 0.0046 min^−1^ with a 500 W Xe arc lamp as light source, indicating that the photocatalytic activity of the BiGdO_3_ sample was significantly improved by using a high-power light source. Figure 15 shows the effect of the light intensity on the photocatalytic degradation efficiency of Direct Red 23 and the first-order kinetic plots for the photocatalytic degradation of Direct Red 23 with different light intensity under visible light irradiation. Similarly, the photocatalytic degradation of Direct Red 23 with a 500 W Xe arc lamp performed better than that with a 250 W Xe arc lamp as shown in Figure 15. The photocatalytic degradation efficiency of Direct Red 23 with a 500 W Xe arc lamp was 60.8% and the first-order rate constant *k* was estimated to be 0.0023 min^−1^ after visible light irradiation of 360 min. While with a 250 W Xe arc lamp as light source, the photocatalytic degradation efficiency of Direct Red 23 was 48.4% and the first-order rate constant *k* was estimated to be 0.0016 min^−1^ after visible light irradiation of 360 min. The main reason of this observation which was reported by previous studies was that higher light intensity provided more photons within a given time, accelerated photolytic reaction ratio, and thus made it possible to achieve maximum degradation yield [101,102,103].

#### 2.2.3. Effect of Coexisting Salts

**Figure 16:**
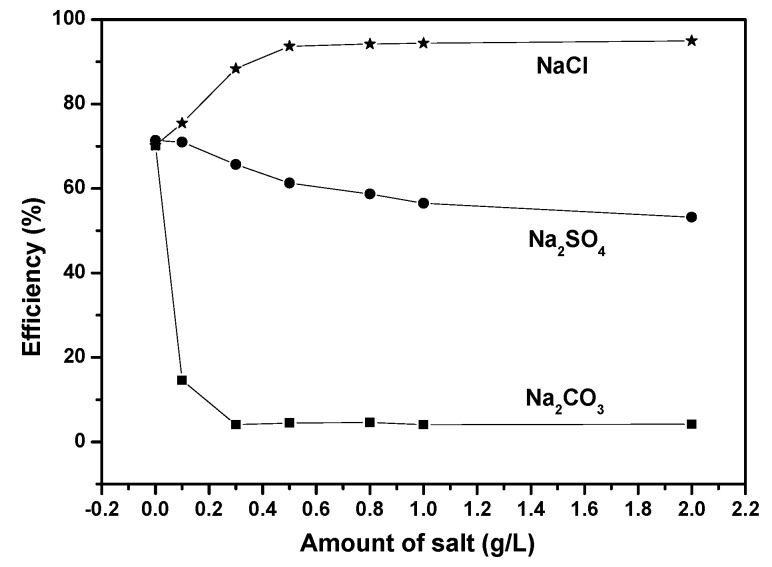
Effect of the coexisting salts on the photocatalytic degradation efficiency of Direct Orange 26.

Composition of actual dye wastewater was very complicated and might contain several salts with different concentrations. In addition, all kinds of salts, which were contained within the dye wastewater, might exert negative or positive influence on the photocatalytic degradation efficiency of dye pollutants. Figure 16 shows the effect of the presence of the coexisting salts on the photocatalytic degradation efficiency of Direct Orange 26. The amount of each salt varied from 0.1 to 2.0 g/L. It can be clearly seen in Figure 16 that the salts had different effect on the photocatalytic degradation efficiency of Direct Orange 26. In case of NaCl, the photocatalytic degradation efficiency of Direct Orange 26 increased with increasing the amount of NaCl and obtained the maximum value with an optimal dosage of 0.5 g/L. On the contrary, both of Na_2_SO_4_ and Na_2_CO_3_ had a detrimental effect on the photocatalytic degradation efficiency of Direct Orange 26. With increasing the amount of Na_2_SO_4_, the degradation efficiency of Direct Orange 26 decreased gently. Similar trend was observed for the photocatalytic degradation of diazo dyes [100,104]. As to Na_2_CO_3_, the degradation efficiency of Direct Orange 26 decreased suddenly when Na_2_CO_3_ of 0.1 g/L was added to the solution. The negative effect could be attributed to the reason that CO_3_^2−^ and SO_4_^2−^ anions inhibited the photocatalytic activity of BiGdO_3_ by trapping ^●^OH or h^+^ in the reaction system [105,106,107,108]. The pH value of the solution varied from 6.2 to 9.1 with the amount of Na_2_CO_3_ which varied from 0 to 2.0 g/L. It can be seen in Figure 13 that the photocatalytic degradation efficiency of Direct Orange 26 gradually decreased when the pH value varied from 4.2 to 10.2. Moreover, CO_3_^2−^ ions addition brought about pH changes that weakened the adsorption of Direct Orange 26 on BiGdO_3_ and inhibited the photocatalytic oxidation [109]. The following Equations (4)–(7) show the scavenging properties of carbonate ion and sulfate ion.

CO_3_^2−^ + ⋅OH → OH^−^ + CO_3_^−^⋅ (4)

HCO_3_^−^ + ⋅OH → H_2_O + CO_3_^−^⋅ (5)

SO_4_^2−^ + h^+^ → SO_4_^−^⋅ (6)

SO_4_^2−^ + ⋅OH → OH^−^ + SO_4_^−^⋅ (7)

Though SO_4_^2−^ played a negative role during the photocatalytic degradation process of Direct Orange 26, the SO_4_^−^^●^ which was produced in Equations (6) and (7) could increase the photocatalytic degradation efficiency of Direct Orange 26 by trapping the photogenerated electrons and/or generating hydroxyl radical (Equations (8) and (9)).

SO_4_^−^⋅ + e^−^ → SO_4_^2−^ (8)

SO_4_^−^⋅ + H_2_O → ⋅OH + SO_4_^2−^ + H^+^ (9)

According to Equations (6) and (7), we acquired a result that generating a certain amount of SO_4_^−^^●^ would consume an equal amount of ^●^OH or h^+^. Although SO_4_^−^^●^ was a strong oxidant, the activity and the amount of product SO_4_^−^^●^ was less than the activity and the amount of ^●^OH or h^+^ [110]. Thus, SO_4_^−^^●^ anions resulted in a mild decrease for the photocatalytic degradation efficiency of Direct Orange 26 compared with CO_3_^2−^ anions. Cl^−^ was also reported to have negative influence on the photocatalytic degradation efficiency of dye pollutants because Cl^−^ was a reducing agent and competed with dye molecules for holes [109,111]. The hole scavenging property for chloride ion was shown in the following Equations (10) and (11).

(10) $${\text{Cl}}^{-}+h^{+}\to \text{Cl}\cdot$$

(11) $$\text{Cl}\cdot +{\text{Cl}}^{-}\to {{\text{Cl}}_{2}}^{-}\cdot$$

While chlorine radicals formed slowly, they were instantaneously turned to chloride radical anions. However, situation was different that the existing of Cl^−^ increased the degradation efficiency of Direct Orange 26 in this experimental case. During the dyeing process of direct dyes, sodium chloride and sodium sulfate were usually utilized to improve the dyeing efficiency of the direct dyes. It was supposed that the existing of Cl^−^ anions or SO_4_^2−^ anions might promote the aggregation of dyes to the surface of the catalysts. Thus, in the case of NaCl, the increase of the degradation efficiency for Direct Orange 26 might be attributed to the effect of good adsorption.

In order to remove negative effects which originated from the formation of sulfate and carbonate ions in the photocatalytic degradation process, we presented the desulfurization system or adjusted the pH value of the solution. Moreover, we also utilized BaCl_2_ to remove sulfate and carbonate ions in the photocatalytic system by using BiGdO_3_ as a photocatalyst under visible light irradiation.

#### 2.2.4. Stability of BiGdO_3_

**Figure 17:**
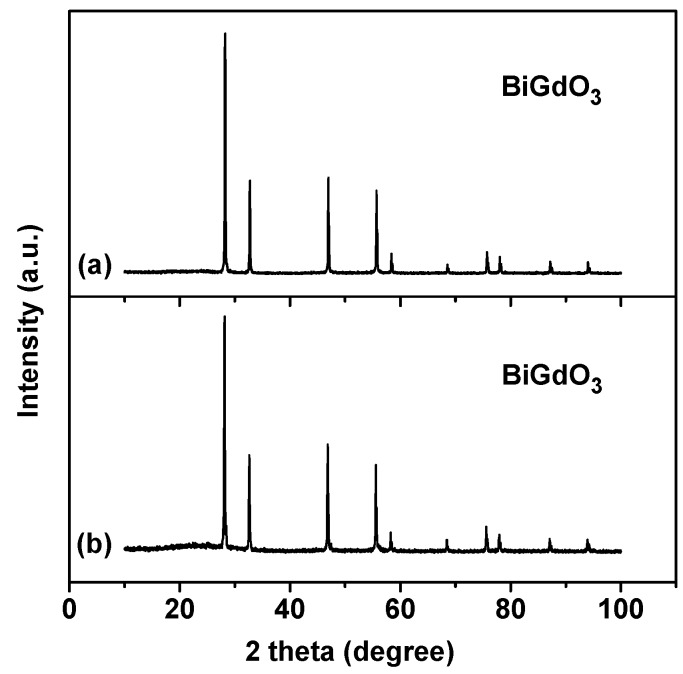
X-ray powder diffraction patterns of every BiGdO_3_ sample: (**a**) the original BiGdO_3_ sample; and (**b**) the BiGdO_3_ sample after photocatalytic degradation of Direct Orange 26.

In order to study the stability of both material and performance for BiGdO_3_ as a visible light catalyst, property characterization of BiGdO_3_ and repeated photocatalytic degradation tests of Direct Orange 26 were carried out. The structure of BiGdO_3_ after photocatalytic degradation of Direct Orange 26 under different conditions was examined by measuring the XRD patterns of every BiGdO_3_ sample. Figure 17 presents the X-ray powder diffraction patterns of all BiGdO_3_ samples that contain the original BiGdO_3_ sample and the BiGdO_3_ sample that was obtained after photocatalytic degradation of Direct Orange 26. It can be seen in the XRD patterns (Figure 17) that the XRD pattern of the BiGdO_3_ sample after the photocatalytic degradation of Direct Orange 26 was the same as that of the original BiGdO_3_ sample.

**Figure 18:**
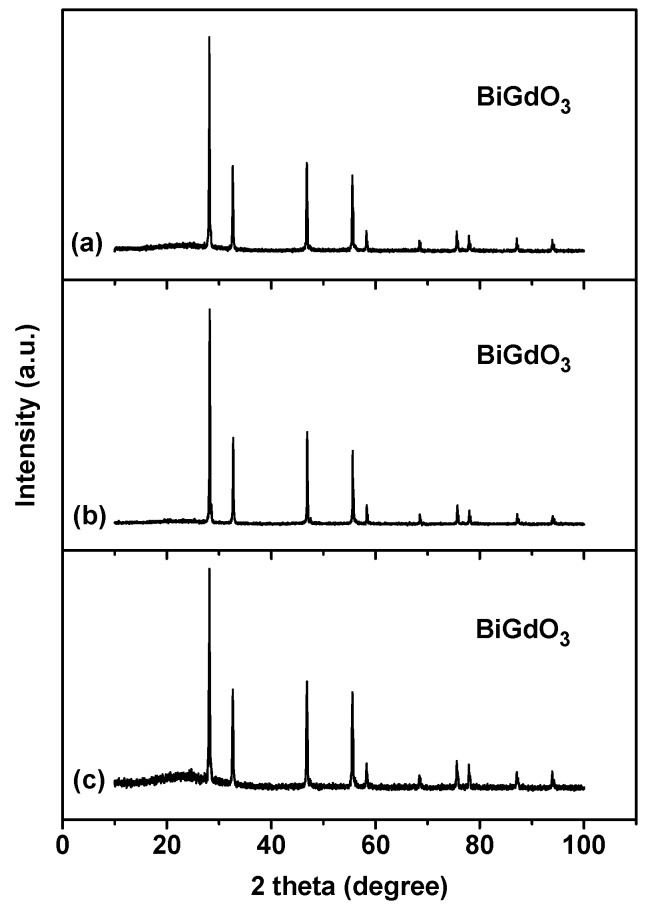
X-ray powder diffraction patterns of all BiGdO_3_ samples after photocatalytic degradation of Direct Orange 26 under different conditions: (**a**) adding salts; (**b**) adding alkali; and (**c**) adding acid.

Figure 18 presents the X-ray powder diffraction patterns of all BiGdO_3_ samples that are obtained after photocatalytic degradation of Direct Orange 26 by adding salts or adding alkali or adding acid. According to the characteristic diffraction peaks as shown in Figure 18, changes could not be observed compared with the peaks of the original BiGdO_3_ sample. Namely, the crystal structure of BiGdO_3_ was stable when acid or alkali or salt was added to the dye solution. The above results clearly indicated the excellent stability of the as-prepared BiGdO_3_.

**Figure 19:**
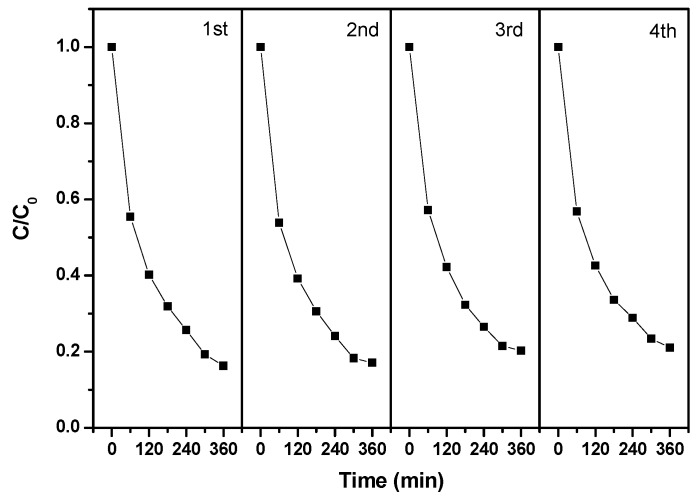
Repeated photocatalytic degradation tests of Direct Orange 26 with BiGdO_3_ as catalyst under visible light irradiation.

Figure 19 presents the repeated photocatalytic degradation tests of Direct Orange 26 with BiGdO_3_ as photocatalyst under visible light irradiation. The photocatalytic degradation of Direct Orange 26 with BiGdO_3_ as photocatalyst under visible light irradiation was repeated four times. It can be seen in Figure 19 that the removal efficiencies of Direct Orange 26 were 82.9%, 79.7% and 78.9% for the second, third and fourth cycles respectively, after visible light irradiation of 360 min. It can be seen in Figure 19 that the activity slightly decreased after the first cycle, which was probably due to the drop of a small amount of BiGdO_3_ particles. Furthermore, the crystal structure of BiGdO_3_ was stable and the morphology of BiGdO_3_ did not change after every repeated cycles. Although the photocatalytic degradation efficiency of Direct Orange 26 for the repeated cycles slightly decreased compared with the photocatalytic degradation efficiency of 83.7% for Direct Orange 26 that was obtained from the first-cycle result, BiGdO_3_ still showed excellent stability and was considered to be an efficient photocatalyst.

**Figure 20:**
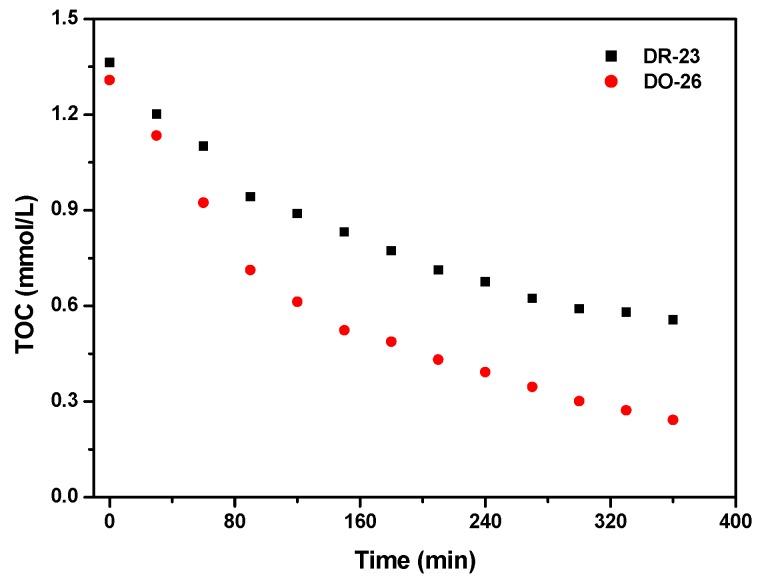
The change of TOC concentration during photocatalytic degradation of Direct Orange 26 and Direct Red 23 with BiGdO_3_ as catalyst under visible light irradiation.

Figure 20 shows the change of TOC concentration during photocatalytic degradation of Direct Orange 26 and Direct Red 23 with BiGdO_3_ as photocatalyst under visible light irradiation. The TOC measurements revealed the disappearance of organic carbon in the Direct Orange 26 solution or Direct Red 23 solution which contained BiGdO_3_. The results indicated that 81.49% of TOC decrease was obtained during photocatalytic degradation of Direct Orange 26 after visible light irradiation for 360 min when BiGdO_3_ was utilized as photocatalyst. In addition, it can also be seen in Figure 20 that 59.19% of TOC decrease was obtained during photocatalytic degradation of Direct Red 23 after visible light irradiation for 360 min with BiGdO_3_ as catalyst. Therefore, the above results showed that Direct Orange 26 was more easily to be mineralized compared with Direct Red 23 with BiGdO_3_ as catalyst under visible light irradiation. According to the results in Figure 8 and Figure 20, it could be found that the photodegradation intermediate products of Direct Orange 26 or Direct Red 23 appeared during the photocatalytic degradation of Direct Orange 26 or Direct Red 23 under visible light irradiation.

**Figure 21:**
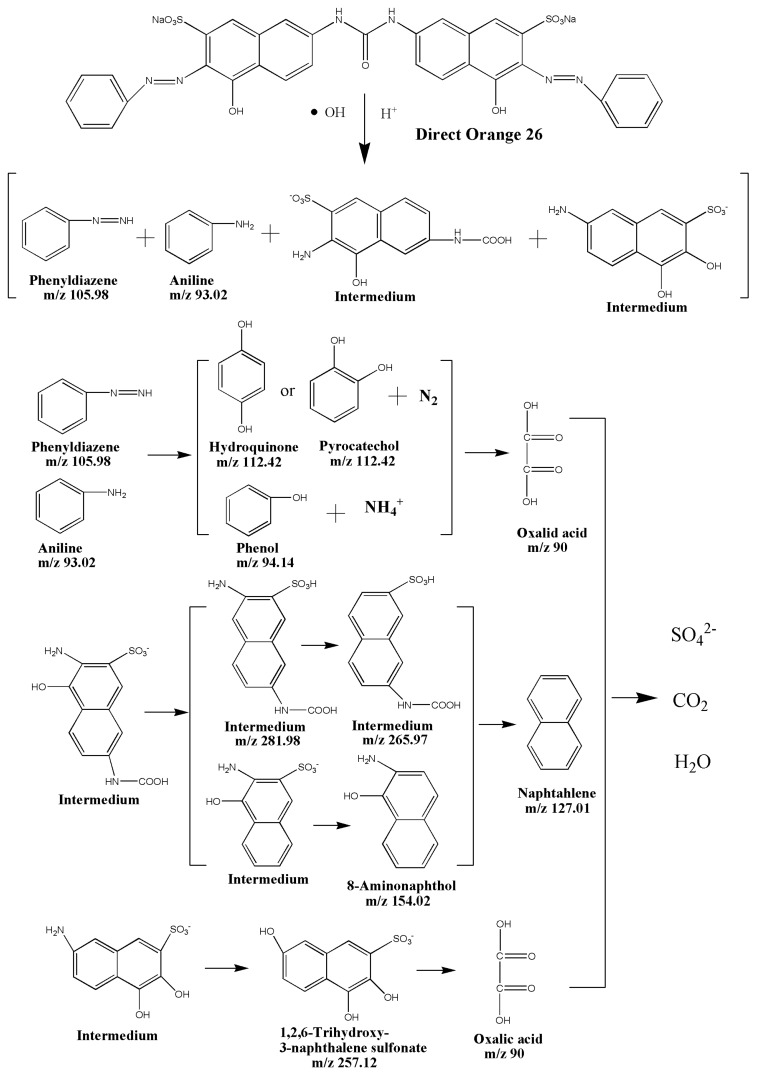
Suggested photocatalytic degradation pathway scheme for Direct Orange 26 under visible light irradiation with BiGdO_3_ as catalyst.

In our experiments, the identified photodegradation intermediate products of Direct Orange 26 with BiGdO_3_ as photocatalyst under visible light irradiation were identified as phenyldiazene (*m*/*z*: 105.98), phenol (*m*/*z*: 94.14), pyrocatechol (*m*/*z*: 112.42), 1,2,6-trihydroxy-3-naphthalene sulfonate (*m*/*z*: 257.12), oxalic acid (*m*/*z*: 90)，8-aminonaphthol (*m*/*z*: 154.02), naphtahlene (*m*/*z*: 127.01), C_11_H_10_O_5_N_2_S (*m*/*z*: 281.98), C_11_H_9_O_5_NS (*m*/*z*: 265.97), and aniline (*m*/*z*: 93.02). Figure 21 shows the suggested photocatalytic degradation pathway scheme for Direct Orange 26 under visible light irradiation with BiGdO_3_ as catalyst. It can be seen in Figure 21 that the chromophore cleavage, opening-ring and mineralization should be the main photocatalytic degradation pathway of Direct Orange 26 in this work. Direct Orange 26 was turned to smaller organic species, and then mineralized together with other organic groups to inorganic products such as CO_2_, SO_4_^2−^, NH_4_^+^, N_2_ and, ultimately, water.

## 3. Experimental Section

### 3.1. Preparation of BiGdO_3_

The BiGdO_3_ sample was prepared by a solid-state reaction method, as was reported previously [112]. Bi_2_O_3_ and Gd_2_O_3_ with purity of 99.99% (Sinopharm Group Chemical Reagent Co., Ltd., Shanghai, China) were utilized as starting materials. Owing to the volatility of Bi_2_O_3_, we finally decided to add 115.6% quantities of Bi_2_O_3_ after 5 experiments. All powders were dried at 200 °C for 4 h before synthesis. In order to synthesize BiGdO_3_, the precursors were stoichiometrically mixed, then pressed into small columns and put into an alumina crucible (Shenyang Crucible Co., Ltd., Shenyang, China). After the raw materials calcining at 750 °C for 10 h, we took the small columns out of the electric furnace, ground the mixed materials and then put into an electric furnace (KSL 1700X, Hefei Kejing Materials Technology Co., Ltd., Hefei, China). After the ground materials were calcined at 850 °C for 10 h, we took the small columns out of the electric furnace, ground the mixed materials and then put them into the electric furnace again. The mixed materials were calcined at 1050 °C for 12 h with an intermediate regrinding process in an electric furnace. Finally, pure BiGdO_3_ catalyst, which presented the color of light orange, was obtained after total grinding. The cost of 100 g Gd_2_O_3_ or Bi_2_O_3_ was 63.2 and 64.5 dollars, respectively, while the synthesis of 100 g pure BiGdO_3_ catalyst totally cost about 71 dollars.

### 3.2. Characterization of BiGdO_3_

The crystal structure of BiGdO_3_ was analyzed by the powder X-ray diffraction method (XRD, XRD 6000, Shimadzu Corporation, Kyoto, Japan) with Cu*K*α radiation (λ = 1.54056 angstrom). The data were collected at 295 K with a step-scan procedure within the range of 2θ = 10°–100°. The step interval was 0.02° and the time per step was 1.2 s. The Bi^3+^ content, Gd^3+^ content and O^2^^−^ content of BiGdO_3_ were determined by X-ray photoelectron spectroscopy (XPS, PHI 5000 Versa Probe, ULVAC-PHI Corporation, Kanagawa, Japan). The chemical composition within the depth profile of BiGdO_3_ was detected by the argon ion denudation method when X-ray photoelectron spectroscopy was utilized. The particle morphology was detected by transmission electron microscope (TEM, JEM-200CX, JEOL Corporation, Tokyo, Japan). The chemical composition of BiGdO_3_ was examined by scanning electron microscope-X-ray energy dispersion spectrum (SEM-EDS, 1530 VP, LEO Corporation, Dresden, Germany). The surface area of BiGdO_3_ particle was measured by the Brunauer-Emmett-Teller method (BET, ASAP 2020, Micromeritics Corporation, Atlanta, GA, USA) with N_2_ adsorption at liquid nitrogen temperature. Totally, 0.6 g BiGdO_3_ was used to measure the surface area of BiGdO_3_ every time. UV-Vis diffuse reflectance spectroscopy measurement of BiGdO_3_ was carried out using an UV-Vis spectrophotometer (DRS, UV-2550, Shimadzu Corporation).

### 3.3. Photocatalytic Experiments

The photocatalytic activity of BiGdO_3_ sample was evaluated by the degradation of Direct Orange 26 (DO-26, C_33_H_22_N_6_Na_2_O_9_S_2_) or Direct Red 23 (DR-23, C_35_H_27_N_7_O_10_S_2_) (AR, Jiangsu Suzhou TLRLHX CO., Ltd., Suzhou, China) in aqueous solution under visible light irradiation (λ > 420 nm). The photocatalytic reaction system was composed of a 500 W Xe arc lamp (Jiangsu Nanjing XJJD Co., Ltd., Nanjing, China), a magnetic stirrer and a cut-off filter (λ > 420 nm, Jiangsu Nanjing XJJD Co., Ltd.). The Xe arc lamp was surrounded by an outer recycling water quartz jacket so that the reaction temperature was maintained at near 25 °C by cooling water in the jacket. The cooling water jacket was surrounded by twelve quartz tubes (50 mL in volume), in which suspensions of direct dyes and photocatalysts were contained. The solution was continuously stirred with a low rate for better light irradiation by the operation of the magnetic stirrer below during the reaction. Prior to light irradiation, the suspension was magnetically stirred in the darkness for 30 min to reach adsorption/desorption equilibrium between the dye and the surface of the catalyst. The reactor was put in the air-saturated conditions to ensure enough oxygen in the reaction solution. The suspension pH values were adjusted to the desired level by using dilute NaOH and HCl, and then the pH values were measured with pH meter (Jiangsu Nanjing ASTKJFZ Co., Ltd., Nanjing, China). During visible light irradiation, one of the quartz tubes was sampled at certain time interval and centrifuged to remove solid particles. The filtrate was analyzed according to the absorption which was measured by a UV-Vis spectrophotometer (UV-2450, Shimadzu Corporation) at a certain wavelength in the UV-Vis spectra of the dyes. Using this method, the conversion percentage of dyes could be obtained at different intervals. The degree of degradation efficiency (*DE*, %) as a function of time was given as follows:

(12) $$DE/\%=\left(C_{0}-C_{t}\right)/C_{0}\times 100\%$$

where *C*_0_ was the initial concentration of the dyes, and *C_t_* was the instant concentration in the sample. The total organic carbon (TOC) concentration was determined with a TOC analyzer (TOC-5000, Shimadzu Corporation).

Intermediate products of Direct Orange 26 were also identified by liquid chromatograph―mass spectrometer (LC-MS, Thermo Quest LCQ Duo, Silicon Valley, CA, USA, HPLC column: β Basic-C18 (150 mm × 2.1 mm × 5 µm), Finnigan, Thermo, Silicon Valley, CA, USA). Here, 20 µL of post-photocatalysis solution was injected automatically into the LC-MS system. The eluent contained 60% methanol and 40% water, and the flow rate was 0.2 mL·min^−1^. MS conditions included an electrospray ionization interface and a capillary temperature of 27 °C with a voltage of 19.00 V, a spray voltage of 5000 V and a constant sheath gas flow rate. The spectrum was acquired in the negative ion scan mode, sweeping the *m/z* range from 50 to 600.

## 4. Conclusions

This work indicated the structural properties, optical properties and photocatalytic activity of BiGdO_3_, which was prepared by a solid state reaction method. Structural characterization of the BiGdO_3_ sample demonstrated that BiGdO_3_ crystallized with the fluorite-type structure, face-centered cubic crystal system and space group *Fm3m 225*. The lattice parameter α for BiGdO_3_ was 5.465 angstrom. UV-Vis diffuse reflectance spectra of BiGdO_3_ displayed that BiGdO_3_ showed a strong optical absorption in the visible light region and the band gap of BiGdO_3_ was estimated to be 2.25 eV. BiGdO_3_ indicated efficient photocatalytic activity for degrading Direct Orange 26 or Direct Red 23 in aqueous solution under visible light irradiation. The photocatalytic degradation efficiency was 83.7% for Direct Orange 26 and 60.8% for Direct Red 23 using BiGdO_3_ as photocatalyst after visible light irradiation of 360 min. The photocatalytic degradation of Direct Orange 26 or Direct Red 23 followed the first-order reaction kinetics, and the first-order rate constant was 0.0046 or 0.0023 min^−1^ with BiGdO_3_ as catalyst. The degradation intermediates of Direct Orange 26 were observed and the possible photocatalytic degradation pathway of Direct Orange 26 under visible light irradiation was provided. The stability of both material and performance of BiGdO_3_ was investigated by material characterization and repeated photocatalytic degradation tests. The results showed that BiGdO_3_ possessed excellent stability and was considered to be an efficient catalyst. BiGdO_3_/(visible light) photocatalysis system was confirmed to be suitable for textile industry wastewater treatment.

The new photocatalyst BiGdO_3_ which was prepared in this present paper and some relevant technical means were beneficial to some engineers or scientists who were engaged in printing and dyeing industry for the purpose of further wastewater treatment. In order to remove organic pollutants from dyes in the actual printing and dyeing wastewater, more improved methods should be implemented as follows: First, a high temperature electric furnace with the maximum sintering temperature of 1400 °C should be purchased. Second, it is important that three-step sintering grinding method be used to synthetize purely single phase BiGdO_3_. During the whole process of preparation of BiGdO_3_, we should pay attention to the increase of heating speed, the control of the maximum sintering temperature and holding time, and the reduction of cooling rate for obtaining the photocatalyst BiGdO_3_ that has perfect crystallinity. Third, for the sake of economizing cost and maximizing the output, we could effectively deal with the actual printing and dyeing wastewater using BiGdO_3_ as a new photocatalyst under the solar light irradiation. Finally, if the concentration of the organic pollutants that exist within actual printing and dyeing wastewater is too high (exceeded 1 mmol/L), we could diluted the printing and dyeing wastewater with pure water to obtain a low concentration of the organic pollutants and then utilized the photocatalyst BiGdO_3_ and the solar light as advanced wastewater treatment technique to degrade the organic pollutants from dyes that exist within the actual printing and dyeing wastewater. All in all, the new photocatalyst BiGdO_3_ that was prepared in this present paper could be applied to the advanced wastewater treatment of low concentration of the organic pollutants that existed within the actual printing and dyeing wastewater.

## Figures and Tables

**Table 1:** Structural parameters of BiGdO_3_.

Atom	*X*	*Y*	*Z*	Occupation Factor
Bi	0.50000	0.50000	0.50000	1.0
Gd	0.00000	0.00000	0.00000	1.0
O	0.25000	0.00000	0.00000	0.5

**Table 2:** Binding energies of Bi 4f, Gd 3d and O 1s in BiGdO_3_.

Elements	Bi 4f^5/2^	Bi 4f^7/2^	Gd 3d^3/2^	Gd 3d^5/2^	O 1s
Binding energy (eV)	163.1	157.8	1218.2	1185.9	528.8
